# Comparison of IPSM 1990 photon dosimetry code of practice with IAEA TRS‐398 and AAPM TG‐51.

**DOI:** 10.1120/jacmp.v10i1.2810

**Published:** 2009-01-14

**Authors:** Silvia Vargas Castrillón, Francisco Cutanda Henríquez

**Affiliations:** ^1^ NW Medical Physics Christie Hospital NHS Foundation Trust Manchester UK

**Keywords:** photon beam dosimetry, code of practice, absorbed dose to water, IPSM, IAEA, AAPM

## Abstract

Several codes of practice for photon dosimetry are currently used around the world, supported by different organizations. A comparison of IPSM 1990 with both IAEA TRS‐398 and AAPM TG‐51 has been performed. All three protocols are based on the calibration of ionization chambers in terms of standards of absorbed dose to water, as it is the case with other modern codes of practice. This comparison has been carried out for photon beams of nominal energies: 4 MV, 6 MV, 8 MV, 10 MV and 18 MV.

An NE 2571 graphite ionization chamber was used in this study, cross‐calibrated against an NE 2611A Secondary Standard, calibrated in the National Physical Laboratory (NPL). Absolute dose in reference conditions was obtained using each of these three protocols including: beam quality indices, beam quality conversion factors both theoretical and NPL experimental ones, correction factors for influence quantities and absolute dose measurements. Each protocol recommendations have been strictly followed. Uncertainties have been obtained according to the ISO Guide to the Expression of Uncertainty in Measurement. Absorbed dose obtained according to all three protocols agree within experimental uncertainty. The largest difference between absolute dose results for two protocols is obtained for the highest energy: 0.7% between IPSM 1990 and IAEA TRS‐398 using theoretical beam quality conversion factors.

PACS number: 87.55.tm

## I. INTRODUCTION

IPSM (Institute of Physical Sciences in Medicine) 1990,^(1)^ AAPM (American Association of Physicist in Medicine) Task Group 51,^(2)^ and the IAEA (International Atomic Energy Agency) Technical Reports Series 398^(3)^ codes of practice are based on the calibration of ionization chambers in terms of absorbed dose to water. This approach is not limited to these codes of practice but is a worldwide trend which includes: German DIN 1997,^(4)^ Japanese Association of Radiological Physics,^(5)^ and the Swiss Society of Radiobiology and Medical Physics.^(6)^ DIN in Germany^(4)^ ^,^ ^(7)^ and IPSM in the United Kingdom^(1)^ ^,^ ^(8)^ were pioneers in the use of standards of absorbed dose to water in their dosimetry protocols. The new standards of calibration of ionization chambers in terms of absorbed dose to water offer the possibility of reducing the uncertainty in the dosimetry of radiotherapy beams, provide a more robust system of primary standards than previous air‐kerma based standards,^(3)^ and allow the use of a more straightforward formalism.

There are similarities and differences among these protocols concerning the choice of quality index, calibration setup, standards of ionization chamber calibration and nomenclature. AAPM TG‐51 and IAEA TRS‐398 have already been discussed in great detail in the literature,^(^ ^9^ ^–^ ^13^ ^)^ particularly concerning the advantages and disadvantages of the recommended photon beam quality indices.^(^ ^14^ ^–^ ^17^ ^)^

### 1A. Calibration

There are different methods of establishing primary standards of absorbed dose to water: ionization chamber (Bureau International des Poids et Mesures in France), water calorimetry (National Research Council in Canada, National Institute of Standards and Technology in the USA), graphite calorimetry (National Physical Laboratory in the UK, Ente per le Nuove Tecnologie, l'Energia e l'Ambiente in Italy, Australian Radiation Protection and Nuclear Safety Agency in Australia, Bundesamt für Eich‐ und Vermessungswesen in Austria, Laboratoire Primaire de Métrologie des Rayonnements Ionisants in France).

The three codes of practice under study here are based on standards of absorbed dose to water; nevertheless, there are some peculiarities regarding the way calibrations are dealt with. The IPSM 1990 protocol is based on the UK National Physical Laboratory ion chamber calibration service, which provides calibration coefficients for a range of beam quality indices.^(^ ^8^ ^,^ ^18^ ^–^ ^21^ ^)^ Their primary standard is a graphite calorimeter, and three ion chambers (NE 2561/2611, NE Technology Ltd, Reading, UK) are used as reference standards in graphite. TG‐51 is based on ion chambers with absorbed dose to water calibration coefficients for Co60 quality $Q_{0}$ and sets of beam quality conversion factors. IAEA TRS‐398 provides the most general and flexible framework for calibration, allowing four very detailed possibilities that include the use of experimental or theoretical beam quality conversion factors (see Section 4.1, IAEA TRS‐398).

IAEA TRS‐398 recommends the use of generic experimental beam quality conversion factors only if they have been determined in a standards laboratory, such as NPL. Values must be obtained from a large sample of ionization chambers and the standard deviation of chamber to chamber variations should be small.^(3)^

Beam quality conversion factors are not included in the IPSM 1990 protocol, however NPL provides experimental values together with $N_{D,w}$ in their most recent calibration certificates (2007). $N_{D,w}$ factors are based on determinations at three or more beam qualities. Based on NPL's vast experience with ion chamber types NE 2561/NE 2611, a generic fit of $k_{Q}$ versus ${\text{TPR}}_{20,10}$ (tissue phantom ratio at 20 cm deep, normalized to 10 cm) is generally used and the ion chamber to be calibrated is checked at several beam qualities. No generic fit is used for other chamber types, and measurements for a full range of beam qualities are carried out instead.

Despite the use of different protocols, all reference dose measurements in this work are traceable to NPL standards. Thus, this comparison is free from any influence related to differences among primary standards and methodologies used in standards laboratories. In a more general situation, when comparing results obtained following different protocols, each institution could be using ionization chambers traceable to different standards and, therefore, dose results for each institution would also include primary standard differences. These differences among standards could lead to differences in calibration factors up to 0.66% according to a BIPM report.^(^ ^3^ ^,^ ^21^ ^–^ ^22^ ^)^

### 1B. Quality Indices and Reference Conditions

The IAEA TRS‐398 and IPSM 1990 recommend ${\text{TPR}}_{20,10}$ as the quality index in order to choose appropriate calibration coefficients $\left(N_{D,w}^{Q}\right)$ or beam quality conversion factors, while AAPM TG‐51 uses $\%\text{dd}{\left(10\right)}_{x}$ (photon component of the percentage depth dose at 10 cm depth).

According to the IPSM 1990 protocol, dose should be measured at 5 cm deep for ${\text{TPR}}_{20,10}$: 0.58–0.75 and Co60; at 7 cm deep for ${\text{TPR}}_{20,10}$: 0.75–0.81. The AAPM TG‐51 and IAEA TRS‐398 recommend a depth of 10 cm, although IAEA TRS‐398 allows 5 cm if ${\text{TPR}}_{20,10}$ is less than 0.7.

In all the protocols, the energy determination is done with a $10\times 10\;{\text{cm}}^{2}$ field at 100 cm SSD. The calibration can be done at the nominal SSD/isocenter location of the accelerator (80 or 100 cm) according to IAEA TRS‐398 and AAPM TG‐51.

### 1C. Beam Quality Conversion Factors

Beam quality conversion factors play an important role in IAEA TRS‐398 ($k_{Q,\text{Qo}}$, when $Q_{0}$ is Co60: $k_{Q}=k_{\text{ggo}}$) and AAPM TG‐51 *(k*) protocols. Both AAPM TG‐51 and IAEA TRS‐398 provide sets of theoretically derived $k_{Q}$ factors for a number of ionization chambers, although IAEA TRS‐398 tables provide factors for a larger number of ionization chambers. IAEA has made an effort to include a wide range of ionization chambers used worldwide in TRS‐398; details on the calculation procedures that lead to these $k_{Q}$ values are given in Appendix B of the IAEA TRS‐398 code of practice, along with uncertainty estimates for each component. The $k_{Q}$ values are based on Bragg‐Gray theory with suitable corrections. The combined standard uncertainty in the values for $k_{Q}$ is 1.0%.^(3)^ ^,^ ^(23)^ ^,^ ^(24)^ TG‐51 concentrates on those chambers used commonly in North America at the time of its publication and includes recommendations on how to use chambers not listed in the protocol.

For TRS‐398 beam quality conversion factors can also be determined experimentally from experimental calibration coefficients for the beam qualities *Q* and $Q_{0}$ using the definition given by Hohlfeld/^7^)

(1) $$k_{Q,Q_{0}}=\frac{N_{D,w}^{Q}}{N_{D,w}^{Q_{0}}}$$

With this method, variations among individual ionization chambers of the same model can be taken into account.

Standards laboratories can take advantage of the cancellation of factors in both calibration coefficients in this equation to reduce the uncertainty in $k_{Q,\text{Qo}}$ (IAEA TRS‐398 notation). Comparison between experimental and theoretical data from IAEA TRS‐398 along with their uncertainties can be found in the comprehensive review by Andreo.^(24)^

A user provided with a set of calibration coefficients $N_{D,w}^{Q}$ for different beam qualities *Q* can compute $k_{\text{Q,Qo}}$ from them, dividing every $N_{D,w}^{Q}$ by $N_{D,w}^{Q0}$, in fact IAEA TRS‐398 recommends this practice. If $k_{\text{Q,Qo}}$ factors are computed following this procedure, their uncertainty has to be derived from the one given for $N_{D,w}^{Q}$ and $N_{D,w}^{Q0}$ in the calibration certificate. This uncertainty value could be larger than the one in standards laboratory measurements, because the user has to assume that $N_{D,w}^{Q}$ and $N_{D,w}^{Q0}$ have independent uncertainty.^(^ ^25^ ^–^ ^27^ ^)^

### 1D. The IPSM 1990 code of practice

There are additional similarities and differences between IPSM 1990 and AAPM TG‐51/IAEA TRS‐398 that are interesting to highlight:

1. IPSM 1990 does not include detailed recommendations about several issues: desired characteristics of field ion chambers, design characteristics of the waterproof sleeve to be used with non‐waterproof field ion chambers, water phantoms, wall thickness when horizontal beams are used, and practical considerations such as limits on leakage current and influence quantities (only generic recommendations are provided).
2. IPSM 1990 and IAEA TRS‐398 use ${\text{TPR}}_{20,10}$ as quality index, while AAPM TG‐51 uses $\%\text{dd}{\left(10\right)}_{x}$. It should be noted that TRS‐398 includes an empirical relationship between ${\text{TPR}}_{20,10}$ and ${\text{PDD}}_{2010}$.
3. IPSM 1990 is based on the calibration of ionization chambers at NPL. Since this code of practice does not include beam quality conversion factors, it would not be possible to use IPSM 1990 for ionization chambers calibrated in standards laboratories which do not provide a calibration at several beam qualities, except for the calibration of a *^60^Co* unit.
4. Neither IPSM 1990 nor AAPM TG‐51 offers any explicit guidelines to estimate measurement uncertainty in a user's beam. IAEA TRS‐398 provides detailed guidelines.
5. IPSM 1990 does not give recommendations about the chamber type to be used when measuring central axis depth dose distributions; specifically, no information is provided about the effective point of measurement of an ionization chamber. TRS‐398 and TG‐51 recommend shifting cylindrical chambers by 0.6 times the inner radius of a cylindrical ion chamber for photon beams.
6. Recombination factors are treated in two different ways in IPSM 1990: the experimental formula by Burns and Rosser^(28)^ (valid only for the NE2561/2611 Secondary Standard [‐200 V polarizing voltage]), and Boag's theory.^(29)^ There is no mention of fits like that by Weinhous and Meli^(30)^, or any approximate relationship such as the ones reported in the other two protocols.

The aims of this paper are to obtain absolute dose results for a set of five photon beam qualities according to the three different codes of practices, and discuss the differences between IPSM 1990 and IAEA TRS‐398/AAPM TG‐51. Attention has been paid to the different methods mentioned in IAEA TRS‐398 and the ones by Rogers^(31)^ and by Kalach and Rogers^(32)^ to obtain ${\text{TPR}}_{20,10}$ and their effect on the value of the beam quality conversion factor and, therefore, in absorbed dose to water. Uncertainty has been evaluated according to ISO Guide to the Expression of Uncertainty in Measurement (GUM) and is discussed below.

## II. MATERIALS AND METHODS

Measurements were performed on two Elekta Synergy (Fleming Way, Crawley, UK) and two Elekta Precise linear accelerators. Photon energies used were 4 MV, 6 M V, 8 M V, 10 MV and 18 MV.

Measurements were carried out with a computer controlled scanner in a large water tank (MP3 beam analyzer, PTW Freiburg, Germany). For central axis depth dose, measurements were performed with a PTW 34001 (Roos type) plane parallel chamber and a 0.125 cm^3^ PTW 31002 cylindrical chamber; both chambers were connected to a PTW Tandem electrometer. The reference setup corresponds to a $10\times 10\;{\text{cm}}^{2}$ field size and SSD=100cm.

IAEA TRS‐398 recommends the use of plane parallel chambers for depth‐ionization measurements. Both chamber types were used in order to compare their measurements since no chamber type is explicitly recommended by IPSM 1990 or by AAPM TG‐51. Cylindrical chambers were shifted 0.6 times their cavity radius in order to place their effective point of measurement at the measuring point, and plane parallel chambers were placed with the centre of the front surface of the chamber air cavity at the measuring point.^(2)^ ^,^ ^(3)^

An NE 2571 graphite chamber connected to a Keithley 35040 Therapy Dosimeter electrometer (Keithley Instruments Inc., Cleveland, OH) was used for absolute dosimetry and ${\text{TPR}}_{20,10}$ measurements. This ionization chamber is not waterproof; therefore it was used with a waterproofing sleeve, meeting the requirements of both IAEA TRS‐398 and AAPM TG‐51 (a PMMA sleeve with a wall not thicker than 1 mm and an air gap of less than 0.2 mm between the chamber and the sleeve).^(2)^ ^,^ ^(3)^

A 0.125 cm^3^ PTW 31002 waterproof thimble chamber was placed inside the phantom at the same depth of the NE 2571 but 4 cm away, and the ratio of both readings was recorded to account for beam output instabilities, following IAEA TRS‐398 recommendations.

### 2A. Quality indices

Several procedures were used for quality index calculation. For 4 MV, 6 MV and 8 MV, $\%\text{dd}{\left(10\right)}_{x}$ was obtained as a measured *%dd(10)*. For higher energy beams the *%dd(10)* needs to be corrected for signals resulting from electron contamination at Dmax. This can be done either by using a generic formula for a typical linac, or by using a 1 mm lead filter to remove the contaminating electrons and replace them with the better known contamination from the lead itself. For 10 MV and 18 MV $\%\text{dd}{\left(10\right)}_{\text{Pb}}$ was measured, placing a lead filter at 52.5 cm distance from water surface and using the equations in the protocols to remove the signal from electrons generated in the lead from the PDD measurements. For the highest energy, the alternative equation for the electron contamination from a typical linac (eq. 15 in TG‐51) was also used to compute $\%\text{dd}{\left(10\right)}_{x}$ from open beam *%dd(10)* (18 MV is the only beam for which it was applicable).

${\text{TPR}}_{20,10}$ was directly measured, keeping the chamber in the same position during the measurements and changing the water level instead. ${\text{TPR}}_{20,10}$ was also computed with the relationship given in TRS‐398 (Section 6.3.1) from *PDD(10)* values, the fit in the publication by Followill^(33)^ which uses the ratio PDD20,10 and the ones proposed by Rogers^(31)^ and Kalach and Rogers^(32)^ using $\%\text{dd}{\left(10\right)}_{x}$.

### 2B. Absolute dosimetry

A graphite chamber NE 2571 (cavity length 24.0 mm, cavity radius 3.2 mm, wall thickness $0.065{\text{gcm}}^{-2}$ and aluminum central electrode) was chosen for these measurements in order to fulfill all three protocol recommendations. The reference instrument in our institution is a NE 2611A Secondary Standard cylindrical chamber (cavity length 9.2 mm, cavity radius 3.7 mm, graphite wall, wall thickness $0.090{\text{gcm}}^{-2}$ and aluminum central electrode), the one recommended by the National Physical Laboratory (Teddington, Middlesex, UK), along with a PTW UNIDOS electrometer. Cross‐calibrations of field chambers take place on a regular basis to ensure traceability to the secondary standard. A regular schedule of constancy checks with Sr‐90 check sources is carried out for both NE 2571 and NE 2611A. All ionization chambers were X‐rayed before and after this study, and were found in proper condition.

The side‐by‐side method was used to cross‐calibrate the NE 2571 ion chamber against the Secondary Standard.^(1)^ Both ion chambers were placed in a perspex phantom meeting the requirements described in IPSM 1990, with two built‐in holes and appropriate perspex inserts to allow chambers to fit in. The inserts can be exchanged along with the chambers. Three sets of measurements were performed; after each, the inserts were exchanged. Cross‐calibrations were carried out at three different depths, corresponding to the reference conditions in the three protocols: 5 cm for 4 MV, 6 MV, 8 MV and 10 MV; 7 cm for 18 MV; 10 cm for all energies. The independence of $N_{D,w}$ with depth has been evaluated in several publications.^(34)^ ^,^ ^(35)^ In our measurements, variations in $N_{D,w}$ between different depths were approximately 0.1% for all energies, well within experimental uncertainty.

Reference conditions for absolute dose measurements were 100 cm SSD, $10\times 10\;{\text{cm}}^{2}$ field size, 10 cm depth in water for IAEA TRS‐398 and AAPM TG‐51. For IPSM 1990, the measurement depths were 5 cm for 4 MV, 6 MV, 8 MV and 10 MV and 7 cm for 18 MV. Corrections for environmental conditions (pressure, temperature) were applied as recommended. Humidity was always within 20% to 70% and temperature within 15° C and 25° C and no humidity correction was necessary. Correction factors (polarity, ion recombination) were measured at the corresponding depth for each beam quality and protocol. No shift was applied, so that in every case the center of the ion chamber was placed at the reference depth according to the different protocols.

Ion recombination correction factors were measured with the two voltage technique according to each protocol. Polarity correction factors were computed as

(2) $$k_{pol}=\frac{\left|M_{+}+M_{-}\right|}{2|M_{-}|}$$

(default polarity is negative). After every change of polarity or absolute voltage, the electrometer and ion chamber were allowed to stabilize for 20 minutes. Four non‐trending readings were taken for each polarity and voltage. The recombination factors were computed according to the quadratic fit by Weinhous and Meli using the approximation given in IAEATRS‐398 and AAPM TG‐51. A factor of 1+X% for a X% decrease in the reading when halving the voltage is the recombination factor recommended by IP SM 1990 and it has been used for the NE 2571 ion chamber.^(1)^

For the secondary standard chamber NE 2611A with –200 V polarizing voltage, NPL and IPSM 1990 protocol provide a formula to obtain the ion recombination factor from the value of the dose per pulse: K=1.0014+0.23p (where p is the dose per pulse in the chamber in cGy.^(28)^) The dose per pulse can be obtained using the PRF (Pulse Repetition Frequency) setting for each beam and the dose rate. This relationship was used for the Secondary Standard chamber as it is the one used at the time of calibration at NPL.

Cross‐calibrations were carried out for the NE 2571 field chamber against the Secondary Standard chamber leading to the corresponding $N_{D,w}$ field for each beam quality. Corrections for influence quantities were taken into account: pressure, temperature, ion recombination, polarity effect, and electrometer calibration. Recombination factors were obtained for the field instrument using the two voltage technique for the field chamber, and using the NPL equation (based on dose per pulse) for the Secondary Standard. Since the NE 2571 ionization chamber was cross‐calibrated with the Secondary Standard for each beam, only $k_{Q}$ factors for the Secondary Standard NE 2611A are needed.

### 2C. Uncertainty

Uncertainties were computed according to the guidelines given in IAEATRS‐398 Appendix B and D, in accordance with ISO GUM.^(25)^ Uncertainties are reported as standard uncertainty (k=1) within this study. In general, a Type A evaluation was performed for each series of raw measurements. Whenever required, uncertainties were composed using the law of propagation of uncertainty where independent uncertainties are added in quadrature.^(25)^

## III. RESULTS & DISCUSSION

Standard uncertainty propagation was used for direct measurements of ${\text{TPR}}_{20,10}$. The fit given in IAEA TRS‐398 (section 6.3.1) for ${\text{TPR}}_{20,10}$ derived from PDD20,10 measurement has a maximum difference with regard to measured data of 0.6% for beam qualities below 50 MV. That means that the Type B standard uncertainty due to the fit in this case is 0.35%, assuming a rectangular probability distribution, and this uncertainty was composed with the Type B uncertainty from the PDD measurements. The same approach was used for the fit by Kalach and Rogers.^(32)^ who report a maximum uncertainty for their fit of measured data on clinical beams of 0.011. On the other hand, we could not find any figure for uncertainty in the quality index relationship given by Followill.^(33)^ although the uncertainty interval (k=2) in the measured values used to design the fit (from nearly 700 machines) is shown in a graph. Thus, only Type B uncertainty from PDD measurements is reported in our results. Finally, no uncertainty estimate was found for the fit by Rogers.^(31)^ Therefore, only Type B uncertainty from PDD measurements is reported in our results for these two fits.

The uncertainty for the AAPM TG‐51 quality index $\%\text{dd}{\left(10\right)}_{x}$ is the Type A one derived from the law of propagation of uncertainty for energies where the use of a lead filter is not recommended. When the quality index is obtained from measurements with a lead filter, an additional Type B uncertainty from the equation is used. Rogers^(36)^ found that by using the fit, the worst case scenario would be that the measured value of $\%\text{dd}{\left(10\right)}_{x}$ is wrong by 0.50%, leading to a standard uncertainty of 0.29%.

Quality indices are used to determine beam quality correction factors, $k_{Q}$. The variation of $k_{Q}$ over the whole range of quality indices in Table I in AAPM TG‐51 is 0.047 ($\%\text{dd}{\left(10\right)}_{x}$ from 50% to 93%),^(2)^ and in Table 6.III in IAEA TRS‐398 it is 0.057 (${\text{TPR}}_{20,10}$ from 0.50 to 0.84).^(3)^ Therefore, quality index uncertainty yields a smaller uncertainty in $k_{Q}$.

**Table 1:** Values for the quality index $\%\text{dd}{\left(10\right)}_{x}$.

*Energy*	$\%\text{dd}{\left(10\right)}_{x}$
4 MV	63.90±0.32
6 MV	67.01±0.33
8 MV	70.02±0.33
10 MV	72.65±0.33
18 MV	80.04±0.39

Concerning beam quality conversion factors $k_{Q}$ in a previous publication, Andreo^(37)^ reports uncertainty estimates of 1.2% to 1.5% for theoretical $k_{Q}$. These values were reduced in TRS‐398 to 1% and reported by Andreo.^(24)^ For $k_{Q}$ factors provided by NPL, we have considered an uncertainty value of 0.7%, based on the report by Sharpe.^(38)^

AAPM quality indices $\%\text{dd}{\left(10\right)}_{x}$ are shown in Table 1. Agreement between depth dose percentage values measured with a plane parallel and a cylindrical ion chamber was found to be within ±0.1% for all beam qualities; PDDs measured with plane parallel chamber were used. As mentioned previously, an additional estimate for $\%\text{dd}{\left(10\right)}_{x}$ for 18 MV has been obtained using equation 15 in TG‐51. The result, 79.74%, is close to the one obtained from $\%\text{dd}{\left(10\right)}_{\text{Pb}}$, 80.04%, and agreement is within experimental uncertainty. The $k_{Q}$ factors computed with each of these values are 0.975 for $\%\text{dd}{\left(10\right)}_{\text{Pb}}=79.74\%$, and 0.976 for $\%\text{dd}{\left(10\right)}_{\text{Pb}}=80.04\%$.

Values for the quality index ${\text{TPR}}_{20,10}$ can be found in Table 2. The largest discrepancy (1.7%) between quality indices obtained with different procedures corresponds to 18 MV for the ${\text{TPR}}_{20,10}$ values obtained using Rogers' and the IAEA relationships from PDD data – which are 0.8% higher and 1.0% lower than measured ${\text{TPR}}_{20,10}$ respectively. Uncertainty for ${\text{TPR}}_{20,10}$ associated with the fits by Followill^(33)^ and Rogers^(31)^ is not available. Their Type B uncertainty due to the PDD measurement is shown in the table, along with values of uncertainty for the IAEA, Kalach and Rogers^(32)^ fits, and the experimental values.

**Table 2:** Values for the quality index ${\text{TPR}}_{20,10}$: directly measured (obtained with Followill's equation from ${\text{PDD}}_{20,10}$, with the equation in Trs‐398 from ${\text{PDD}}_{10}$, and with the fits given by Rogers and Kalach).

*Energy*	*TPR Measured 20,10*	*TPR Followill 20,10*	*TPR IAEA 20,10*	*TPR Rogers 20,10*	*TPR Kalach 20,10*
4 MV	0.6381±0.0019	0.6355±0.0068	0.6347±0.0042	0.6443±0.0032	0.6394±0.0073
6 MV	0.6690±0.0018	0.6710±0.0065	0.6694±0.0040	0.6765±0.0030	0.6745±0.0072
8 MV	0.7082±0.0018	0.7102±0.0078	0.7000±0.0046	0.7050±0.0036	0.7048±0.0074
10 MV	0.7303±0.0019	0.7314±0.0077	0.7221±0.0044	0.7278±0.0033	0.7280±0.0072
18 MV	0.7782±0.0017	0.7812±0.0075	0.7714±0.0038	0.7845±0.0025	0.7798±0.0068

**Figure 1:**
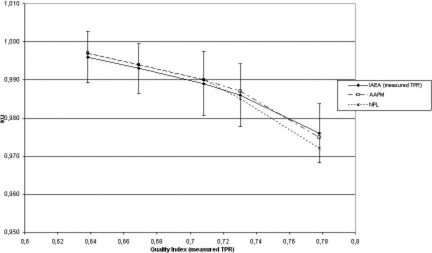
Beam quality conversion factors $k_{Q}$ as a function of nominal energy. Graphs are shown for theoretical $k_{Q}$ from IAEA TRS‐398 (using measured ${\text{TPR}}_{20,10})$, AAPM TG‐51 and experimental $k_{Q}$ from NPL.

Results for beam quality conversion factors (NE 2611) for the five beam qualities, according to theoretical tables in IAEA TRS‐398 and AAPM TG‐51 and experimental results from NPL, are plotted in Figure 1. Differences in the quality index ${\text{TPR}}_{20,10}$ obtained by different procedures would lead to a $k_{Q}$ difference of 0.4% for the case of TRS‐398 theoretical $k_{Q}$ factors and of 0.6% for experimental $k_{Q}$ (Tables 3 and 4). Differences between experimental and theoretical $k_{Q}$ are found to be larger for the highest energies as reported by Andreo^(24)^ but the differences are well within the error bars for all energies.

**Table 3:** Theoretical $k_{Q}$ factors from IAEA TRS‐398 and AAPM TG‐51 tables, computed with each of the ${\text{TPR}}_{20,10}$ obtained by different methods.

	$k_{Q}({\text{TPR}}_{20,10}^{\text{Meas}.)}$	$k_{Q}({\text{TPR}}_{20,10}^{\text{Foll}.)}$	$k_{Q}\left({\text{TPR}}_{20,10}^{\text{IAEA}}\right)$	$k_{Q}({\text{TPR}}_{20,10}^{\text{Rog}.)}$	$k_{Q}({\text{TPR}}_{20,10}^{\text{Kal}.)}$	$k_{Q}(\%\text{dd}{\left(10\right)}_{x})$
4 MV	0.996	0.996	0.996	0.995	0.995	0.997
6 MV	0.993	0.993	0.993	0.992	0.992	0.994
8 MV	0.989	0.989	0.990	0.989	0.990	0.990
10 MV	0.986	0.986	0.988	0.987	0.987	0.987
18 MV	0.976	0.975	0.978	0.974	0.975	0.975

**Table 4:** Experimental $k_{Q}$ factors from NPL calibration certificate for the ion chamber NE2611, computed with each of the ${\text{TPR}}_{20,10}$ obtained by different methods.

*Energy*	$k_{Q}({\text{TPR}}_{20,10}^{\text{Meas}.)}$	$k_{Q}({\text{TPR}}_{20,10}^{\text{Foll}.)}$	$k_{Q}\left({\text{TPR}}_{20,10}^{\text{IAEA}}\right)$	$k_{Q}({\text{TPR}}_{20,10}^{\text{Rog}.)}$	$k_{Q}({\text{TPR}}_{20,10}^{\text{Kal}.)}$
4 MV	0.997	0.997	0.997	0.996	0.997
6 MV	0.994	0.994	0.994	0.993	0.994
8 MV	0.990	0.989	0.991	0.990	0.990
10 MV	0.985	0.985	0.987	0.986	0.986
18 MV	0.972	0.971	0.975	0.969	0.971

Polarity factors and ion recombination factors were obtained at the corresponding depths (10 cm for IAEA TRS‐398 and AAPM TG‐51, 7 cm and 5 cm for IPSM 1990). The maximum difference for ion recombination factors, 0.2%, has been found between IPSM 1990 and AAPM TG‐51 for the 18 MV beam (1.008 for TG‐51 and 1.010 for IPSM 1990). Despite the difference in depth, recombination factors for IPSM are within 0.1% of the values for the other two protocols and the remaining beam qualities. The differences in recombination factors between IAEA TRS‐398 and AAPM TG‐51, measured at the same depth, are 0.1% or less for all beam qualities. Uncertainty in recombination factor determination is below 0.2% when care is taken to ensure the measuring system is given adequate time to settle after changes in polarizing voltage. The polarity factor is identical for TRS‐398 and TG‐51 and is less than 0.1% different for IPSM 1990, and its uncertainty is below 0.3%.

Absolute absorbed dose to water in reference conditions has been finally computed in four different ways, using the common formalism given by:

(3) $$D(z_{ref})=N_{D,w}^{Q,field}\cdot M^{*}$$

where $M^{*}$ is the reading corrected for all influence quantities. It has been divided by the appropriate PDD value to obtain the absorbed dose at maximum. The four methods are:

1. IAEA TRS‐398, with $N_{D,w}^{C_{0}}$ and experimental beam quality conversion factors for the secondary standard provided by NPL.
2. IAEA TRS‐398, with $N_{D,w}^{Q}$ factors computed applying theoretical $k_{Q}$ factors (from IAEA TRS‐398 tables) to the $N_{D,w}^{C_{0}}$ provided by NPL.
3. AAPM TG‐51, with $N_{D,w}^{Q}$ factors computed applying theoretical $k_{Q}$ factors (from AAPM TG‐51 tables) to the $N_{D,w}^{C_{0}}$ provided by NPL.
4. IPSM 1990, measuring at 5 or 7 cm deep instead of 10 cm deep, and using $N_{D,w}^{Q}$ calibration coefficients from the NPL calibration certificate.

The ratios of absorbed dose obtained with IAEA TRS‐398 and AAPM TG‐51 to IPSM 1990 were found to be close to 1.005 (Table 5). Huq^(9)^ reported overall differences between IAEA TRS‐398 and AAPM TG‐51 of 0.2% with a single case of 0.3% for 18 MV. Our results are in agreement with these values for these two codes of practice (AAPM TG‐51 results compared with IAEA TRS‐398 computed from theoretical beam quality correction factors). The work by Huq does not address the case of experimental beam quality correction factors. The reason for the differences between the first and second column in Table 5 (IAEA absolute dose results using theoretical and experimental $k_{Q}$) is related to the differences in the beam quality correction factors (see Tables 3 and 4).

**Table 5:** Absolute dose quotients of IAEA TRS‐398/IPSM 1990 and AAPM TG‐51/IPSM 1990. For IAEA TRS‐398, results for experimental and theoretical $k_{Q}$ factors are included.

	$D_{m}^{\text{IAEA}}(k_{Q}^{\text{exp}})/D_{m}^{\text{IPSM}}(N_{D,w})$	$D_{m}^{\text{IAEA}}(k_{Q}^{\text{theo}})/D_{m}^{\text{IPSM}}(N_{D,w})$	$D_{m}^{\text{AAPM}}(k_{Q})/D_{m}^{\text{IPSM}}(N_{D,w})$
4 MV	1.005±0.022	1.004±0.026	1.005±0.022
6 MV	1.004±0.021	1.003±0.026	1.004±0.023
8 MV	1.004±0.028	1.003±0.031	1.004±0.029
10 MV	1.003±0.027	1.004±0.032	1.005±0.028
18 MV	1.003±0.028	1.007±0.032	1.006±0.032

## IV. CONCLUSIONS

IPSM 1990 photon dosimetry code of practice has been compared with AAPM TG‐51 and IAEA TRS‐398, and the similarities and differences between IPSM 1990 and AAPM TG‐51/IAEA TRS‐398 have been discussed.

Uncertainties have been computed for all experimental results according to the ISO Guide for the Expression of Uncertainty in Measurement recommendations^(25)^. Absorbed dose to water determinations according to the three protocols agree within experimental uncertainty, and this uncertainty is similar to the one reported in IAEA TRS‐398. The maximum difference in absorbed dose to water determination is obtained for 18 MV: IPSM 1990 result is 0.7% lower than the IAEA TRS‐398 one using its theoretical beam quality conversion factors. This maximum difference is mainly related to the use of experimental beam quality conversion factors for IPSM 1990 and theoretical ones for IAEA TRS‐398 (Fig. 1). It must be stressed that this comparison has been performed with a single calibration from NPL and that the use of different protocols and calibrations traceable to different standards laboratories will add further differences increasing or lowering results (see Section 2.2 and Table 2.2 in IAEA TRS‐398).

## ACKNOWLEDGEMENTS

The authors are grateful to Huey Yang for his careful revision of the manuscript and to Lisa Rodan for grammar revision.

## Supporting information

Supplementary Material
