# Supplementary figures and images for: Comparison of IPSM 1990 photon dosimetry code of practice with IAEA TRS‐398 and AAPM TG‐51
Source: J Appl Clin Med Phys. 2009 Jan 14;10(1):136–46. doi: 10.1120/jacmp.v10i1.2810 (PMC5720500; doi:10.1120/jacmp.v10i1.2810)

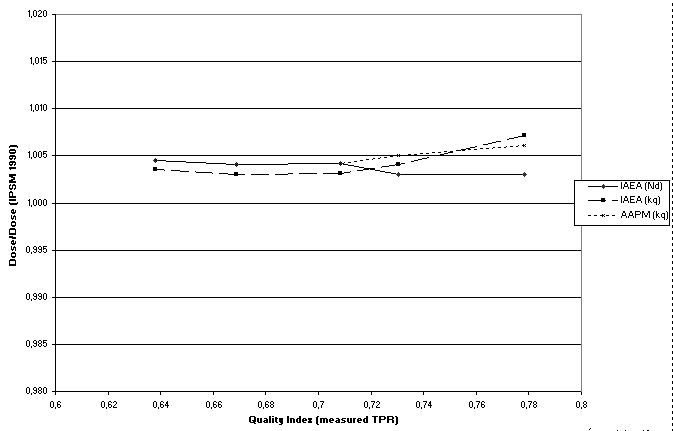

Supplement: Supplementary file 1 — Supplementary Material [file ACM2-10-136-s001.jpg]
